# Histomorphometry indicates distinct contributions of myofibroblast activation and sinusoidal capillarization to portal hypertension

**DOI:** 10.1007/s12072-026-11133-0

**Published:** 2026-07-27

**Authors:** Thomas Sorz-Nechay, Benedikt S. Hofer, Philipp Königshofer, Alexis Treitler, Georg Semmler, Benedikt Simbrunner, Vlad Taru, Katharina Bonitz, Henriette Horstmeier, Georg Kramer, Kerstin Zinober, Katharina Regnat, Barbara Neudert, Christopher C. Kaltenecker, Behrang Mozayani, Renate Kain, Michael Trauner, Mattias Mandorfer, Philipp Schwabl, Thomas Reiberger

**Affiliations:** 1 Division of Gastroenterology and Hepatology, Department of Medicine III, Medical University of Vienna, Währinger Guertel 18-20, 1090 Vienna, Austria; 2 Christian Doppler Laboratory for Portal Hypertension and Liver Fibrosis, Medical University of Vienna, Vienna, Austria; 3 CeMM Research Center for Molecular Medicine of the Austrian Academy of Sciences, Vienna, Austria; 4 Clinical Research Group MOTION, Medical University of Vienna, Vienna, Austria; 5 Department of Pathology, Medical University of Vienna, Vienna, Austria

**Keywords:** Portal hypertension, Chronic liver disease, Cirrhosis, Liver fibrosis, Histology, Biomarker, Pathophysiology

## Abstract

**Background and aims:**

Portal hypertension (PH) is a severe consequence of cirrhosis and the primary driver of hepatic decompensation. We aimed to investigate the link between histological myofibroblast activation (alpha-smooth-muscle-actin: αSMA) and sinusoidal capillarization (CD34) markers to PH severity in patients with chronic liver disease (CLD).

**Methods:**

Patients with CLD underwent hepatic venous pressure gradient (HVPG) measurement and liver biopsy. Liver samples were stained by picrosirius-red for collagen proportionate area (CPA) and for αSMA/CD34. Quantitative histomorphometry was performed on full-slide scans.

**Results:**

102 patients (63.7% male, median age 56.4 years, mainly steatotic liver disease: 58.8%) were included. The median HVPG was 13 [10–18] mmHg and severe PH (HVPG ≥ 16 mmHg, PH16) was found in 35.3%. Median liver-stiffness was 30.6 [19.1–57.6] kPa. In PH16 patients, fibrosis (CPA: 28.39 vs 17.72%), myofibroblast activation (αSMA: 10.84 vs 3.75%), and capillarization (CD34: 5.45 vs 2.67%) were significantly more pronounced (all p < 0.001 vs. no PH16). CD34 showed a higher correlation to HVPG (rho = 0.505) than CPA (rho = 0.472) and αSMA (rho = 0.347, all p < 0.001). CD34 expression was independently correlated with enhanced liver fibrosis (ELF) test and von Willebrand factor (VWF). When partially adjusting for fibrosis severity, patients with less severe fibrosis and high αSMA or CD34 had a trend for higher HVPG, whereas in intermediate fibrosis, only CD34-high patients trended higher HVPG.

**Conclusion:**

CPA, CD34 and αSMA expression all correlate with PH severity. CD34 represents a histologic sinusoidal capillarization marker that correlates with circulating VWF and ELF. αSMA and CD34 represent potential histologic markers of PH beyond the static component.

**Graphical abstract:**

**Figure:**
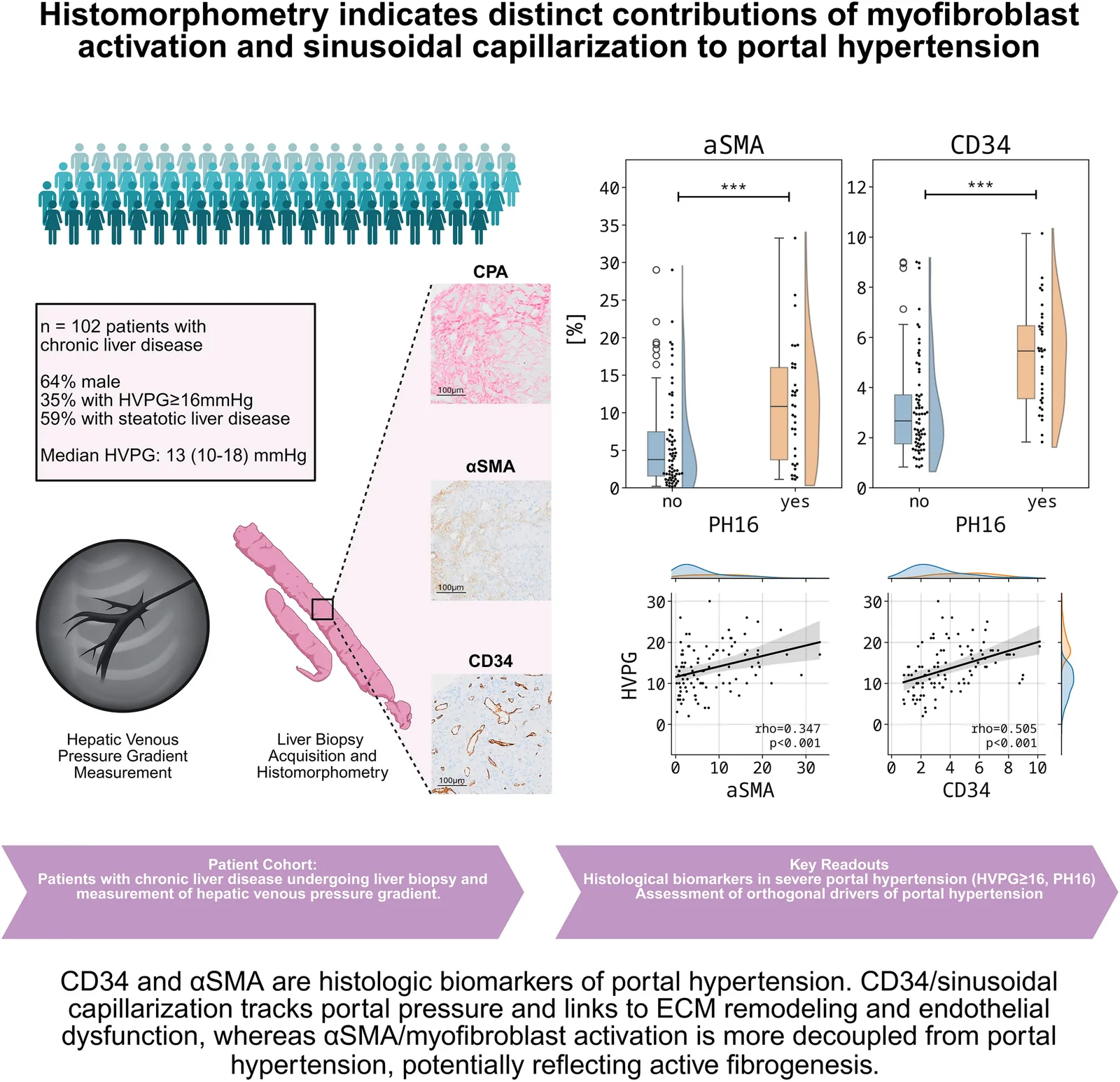

**Supplementary Information:**

The online version contains supplementary material available at https://doi.org/10.1007/s12072-026-11133-0.

## Introduction

Cirrhosis represents a global health challenge and a large socioeconomic burden, causing 2 million deaths annually [1]. Portal hypertension (PH), a severe consequence of cirrhosis, is a driver of morbidity and mortality in patients [2].

Liver damage prompts the initiation of an inflammatory cascade, which leads to immune cell infiltration and release of pro-inflammatory mediators [3]. Consequently, quiescent hepatic stellate cells (HSC) trans-differentiate into myofibroblasts, which become contractile, excessively produce collagen, and express alpha-smooth-muscle actin (αSMA) [3]. Liver sinusoidal endothelial cells (LSEC) become impaired and undergo capillarization [4], indicated by expression of CD34 [5]. The interplay of architectural disruption, endothelial dysfunction and inflammation leads to increased intrahepatic resistance and PH.

The gold-standard in PH assessment is the measurement of the hepatic venous pressure gradient (HVPG) [6]. An HVPG increase to ≥ 10 mmHg defines clinically significant portal hypertension (CSPH), which indicates risk of progressing to hepatic decompensation. A further rise in HVPG to ≥ 16 mmHg (PH16) indicates an immanent risk of developing a decompensating event and to suffer complications after abdominal surgery [7, 8]. Importantly, approximately two thirds of PH severity are explained by structural changes like liver fibrosis, which is termed the static component of PH. The remaining one third constitutes the dynamic component [9]. The dynamic component has been associated with vasoconstriction (e.g. through myofibroblast contraction), endothelial dysfunction and inflammation [10–14]. It is more readily reversible and can be partially modulated via non-selective beta blockers [9].

Traditionally, cirrhosis has been diagnosed through liver biopsy. Evaluation of liver biopsies yields valuable insights into the underlying etiology and fibrosis severity, while also offering prognostic information [15]. Numerous semi-quantitative histopathological fibrosis scores have been developed [16], however, they require assessment by expert pathologists, are limited by inter-observer variability [17] and do not accurately reflect the dynamic nature of liver fibrogenesis. Thus, more objective approaches were developed to improve the diagnostic utility of liver biopsies.

Collagen proportionate area (CPA) assessment via digital histomorphometry provides a continuous parameter which more accurately reflects the amount of fibrosis and the static component of PH. CPA has been used to predict CSPH, hepatic decompensation and liver-related death in compensated advanced chronic liver disease [18–20]. CPA also correlates with various clinical parameters of liver fibrosis and portal hypertension, such as enhanced liver fibrosis (ELF) test and HVPG [21, 22]. However, dynamic histological biomarkers of PH, such as αSMA for the assessment of myofibroblast activation and contractility [3] and CD34 for the assessment of sinusoidal capillarization [5], remain underexplored.

While αSMA and CD34 are considered markers of myofibroblast activation and sinusoidal capillarization, their relationship to PH severity has not been systematically characterized. We therefore aimed to elucidate the pathomechanistic links of hepatic myofibroblast activation/contractility and sinusoidal capillarization/vascular remodeling - as evaluated via quantitative histomorphometry of αSMA and CD34 - to PH severity in a cross-sectional cohort of patients with chronic liver disease (CLD).

## Materials and methods

### Study design and patient cohort

This single-center study included adult patients with chronic liver disease undergoing HVPG measurements with concomitant liver biopsies (acquired within ± 3 months of HVPG) between 2014 and 2024 at the Vienna Hepatic Hemodynamic Lab of the Medical University of Vienna. All patients signed an informed consent form for the prospective Vienna Cirrhosis Study (ethics number: 1262/2017, clinicaltrials.gov number: NCT03267615).

Patients were excluded from the study based on the following criteria: Current or prior hepatocellular carcinoma, liver metastasis, portal vein thrombosis, transjugular intrahepatic portosystemic shunt or liver transplantation.

### Hemodynamic assessment and clinical data

HVPG was measured according to a standard operating procedure [6] that adheres to national [23, 24] and international [2] recommendations. Briefly, a catheter was placed via transjugular route and HVPG was calculated based on triplicate measurements of wedged hepatic venous pressure subtracted from free hepatic venous pressure. Blood was withdrawn within the setting of the HVPG measurement using a central venous catheter. Liver specimens were obtained either via transjugular or percutaneous biopsy, following clinical standard operating procedures. Liver stiffness measurement (LSM) was conducted via vibration-controlled transient elastography on a FibroScan device (Echosens, Paris, France). Clinical metadata were collected from electronic patient records.

### Histological staining and digital histomorphometry

All liver biopsy specimens were formaldehyde-fixed (7.5%) and paraffin-embedded. Unstained tissue sections were obtained via microtomy at 2 μm thickness. For CPA analysis, slides were stained using picrosirius-red staining and fast-green counterstaining. For αSMA and CD34, slides were stained immunohistochemically (αSMA: mouse monoclonal antibody to Actin, Smooth Muscle [1A4], #760-2833 IVD, ready-to-use, CellMarque, California, USA; CD34: mouse monoclonal antibody to CD34 [QBEnd/10], #NCL-L-END, dilution 1:50, Leica Biosystems, Nussloch, Germany) after enzymatic antigen retrieval and visualized by automated immunohistochemistry with a Novolink™ Polymer Detection system on an Immunostainer Benchmark Ultra (Ventana/Roche). Following dehydration in ascending concentrations of ethanol and immersion in xylene, all slides were covered in hydrophobic mounting medium and digitized in high-resolution (40× objective, 0.137 μm/pixel) using the Olympus SLIDEVIEW VS200 research slide-scanner (EVIDENT Europe GmbH, Hamburg, Germany). Digital histomorphometry was performed using a customized computational pipeline developed in Python (version 3.10.14). Each histological biomarker (CPA, αSMA, CD34) was quantified as the proportion of positively stained area relative to the total tissue area, expressed in percentages. Briefly, images were converted to Hue-Saturation-Lightness color space to determine the tissue area via tissue segmentation, followed by conversion to Hue-Saturation-Value color space to determine the positive area of the histological biomarker via color-based segmentation. A detailed description of the histological processing and analysis protocols is provided in Supplementary Materials.

### Analysis of fibrosis-independent pathomechanistic axes

To identify pathophysiological drivers of PH beyond static fibrosis - reflected histologically by CPA and clinically by LSM - we performed three analyses. First, we stratified patients based on a linear regression of HVPG on CPA (99% confidence interval, CI). Patients whose HVPG exceeded the upper bound of the 99% confidence interval were classified as higher-than-expected HVPG (HVPG_high_), while the remaining patients were classified as expected HVPG (HVPG_exp_). We then compared αSMA, CD34, ELF, C-reactive protein (CRP), interleukin 6 (IL6), albumin, cholinesterase and bilirubin between both strata. Second, to assess effects of αSMA and CD34 on HVPG in a setting where we additionally control for fibrosis severity, we stratified patients into three cohorts based on CPA tertiles (i.e. the 33rd and 67th percentile). Within each cohort, we sub-stratified based on within-tertile median αSMA and median CD34 and compared HVPG values.

Lastly, we performed partial correlation analysis to identify pathomechanistic axes of the histological biomarkers in a data-driven approach. For each parameter of interest, variables were rank-transformed and a multivariate linear regression was fitted using a defined covariate set and the residuals were extracted representing the proportion of the ranked value unexplained by those covariates. Residual-residual correlations were then computed to reveal shared pathomechanistic axes independent of the covariates. We defined three covariate sets, each including HVPG and two histological biomarkers in alternation, such that the remaining histological biomarker was treated as a parameter of interest. For example, when covariates were HVPG, αSMA and CPA, residuals were computed for CD34 and all clinical parameters and correlated.

### Statistical analysis

All statistical analyses were performed in Python (version 3.12.3) using scipy (version 1.13.1) and scikit_posthocs (version 0.11.4) packages. All variables were tested for normality. Normally distributed variables were analyzed through Student’s t-test. Otherwise, Mann–Whitney U-test was used. Frequency analysis was performed using chi-squared or Fisher’s Exact test if the expected frequency for a group was < 5. Correlation analysis was performed using Spearman’s rho coefficient. Partial correlation analysis was performed via the pingouin (version 0.5.5) package to identify the independent correlation (Spearman’s rho coefficient) of histological biomarkers with other clinical variables. Clinical utility of histological biomarkers was tested using logistic regression (sklearn package, version 1.5.1) to predict presence of CSPH or PH16. Performance was estimated using AUROC and optimal cut-offs were determined with Youden’s index. All descriptive values represent medians, with ranges indicating the 25th to 75th percentile, formatted as: median [25th–75th percentile].

## Results

### Patient characteristics (Table 1)

**Table 1:** Cohort characteristics

Variable	HVPG < 16 mmHg	HVPG ≥ 16 mmHg	p value	Full cohort
	n = 66 (64.7%)	n = 36 (35.3%)	–	n = 102 (100.0%)
Sex				
Male	41 (62.1%)	24 (66.7%)	0.810	65 (63.7%)
Female	25 (37.9%)	12 (33.3%)		37 (36.3%)
Age [years]	57.4 (50.4—66.7)	56.0 (47.9—60.0)	0.144^a^	56.4 (49.5—64.7)
BMI [kg/m^2^]	27.0 (23.0—30.5)	27.8 (24.3—30.2)	0.421^a^	27.4 (23.6—30.5)
Etiology				
SLD	36 (54.5%)	24 (66.7%)	0.347	60 (58.8%)
Other	21 (31.8%)	10 (27.8%)		31 (30.4%)
Viral	9 (13.6%)	2 (5.6%)		11 (10.8%)
Fibrosis stage				
≤ F3	27 (40.9%)	4 (11.1%)	0.004	31 (30.4%)
F4	39 (59.1%)	32 (88.9%)		71 (69.6%)
Varices				
Yes	24 (36.4%)	23 (63.9%)	0.011	47 (46.1%)
CTP stage^b^				
A	44 (66.7%)	9 (25.0%)	< 0.001	53 (52.0%)
B–C	22 (33.3%)	26 (72.2%)		48 (47.1%)
Decompensated				
No	42 (63.6%)	6 (16.7%)	< 0.001	48 (47.1%)
Yes	24 (36.4%)	30 (83.3%)		54 (52.9%)
HVPG [mmHg]	10 (8—13)	19 (17—21)	< 0.001^a^	13 (10—18)
LSM [kPa]	21.9 (16.6—34.4)	58.6 (35.6—74.7)	< 0.001^a^	30.6 (19.1—57.6)
ELF [score]	11.02 (10.33—12.34)	12.53 (11.95—13.17)	< 0.001^a^	11.65 (10.81—12.67)
Platelets [G/L]	124 (88—192)	114 (79—161)	0.302^a^	121 (86—180)
VWF [%]	273 (204—327)	330 (273—420)	< 0.001^a^	287 (225—369)

All metric variables were tested for normality using Shapiro–Wilk test (if n > 50) or normal-test (if n < 50). Metric variables are represented as median (Q1–Q3)

*BMI* body-mass index, *CTP* Child-Turcotte-Pugh, *ELF* enhanced liver fibrosis score, *HVPG* hepatic venous pressure gradient, *LSM* liver stiffness measurement, *SLD* steatotic liver disease, *VWF* von-Willebrand factor

^a^p values derived from Mann–Whitney-U test (metric)

^b^CTP Stage was missing for one patient

In total, 102 patients were included in the study (Table 1), with a median patient age of 56.4 [49.5–64.7] years; 63.7% (n = 65/102) were male. The most common etiology was steatotic liver disease (n = 60/102, 58.8%; consisting of: alcohol-related liver disease: n = 32/102, 31.4%; metabolic dysfunction-associated steatotic liver disease: n = 21/102, 20.6%; and MetALD: n = 7/102, 6.9%). Other etiologies included cholestatic liver disease (n = 14/102, 13.7%), cryptogenic liver disease (n = 6/102, 5.9%), viral liver disease (n = 11/102, 10.8%) and mixed etiologies (n = 11/102, 10.8%). The median HVPG value was 13 [10–18] mmHg, and 35.3% (36/102) had PH16, whereas 76.5% (78/102) had CSPH. Patients who presented with PH16 at HVPG measurement more frequently had decompensated disease (n = 30/36 with PH16, 83.3% vs. n = 24/66 without PH16, 36.4%; p < 0.001). Most patients had histologically diagnosed cirrhosis (n = 71/102, 69.6%), with nearly half of all cirrhotic patients having manifest PH16 (PH16: n = 32/71, 45.1%). In patients with non-cirrhotic histology (i.e. stages ≤ F3: 31/102, 30.4%), PH16 was almost completely absent (PH16: 4/31, 12.9% vs. no PH16: 27/31, 87.1%; p = 0.004). Patients did not significantly differ in age (p = 0.144) or body mass index (p = 0.421), but LSM was significantly higher in the PH16 group (p < 0.001). ELF, a marker of extracellular matrix remodeling, and von Willebrand factor (VWF), a marker of endothelial dysfunction, were also significantly elevated in patients with PH16 (p < 0.001 and p = 0.001, respectively).

### Portal hypertension and histological biomarker expression

**Fig. 1:**
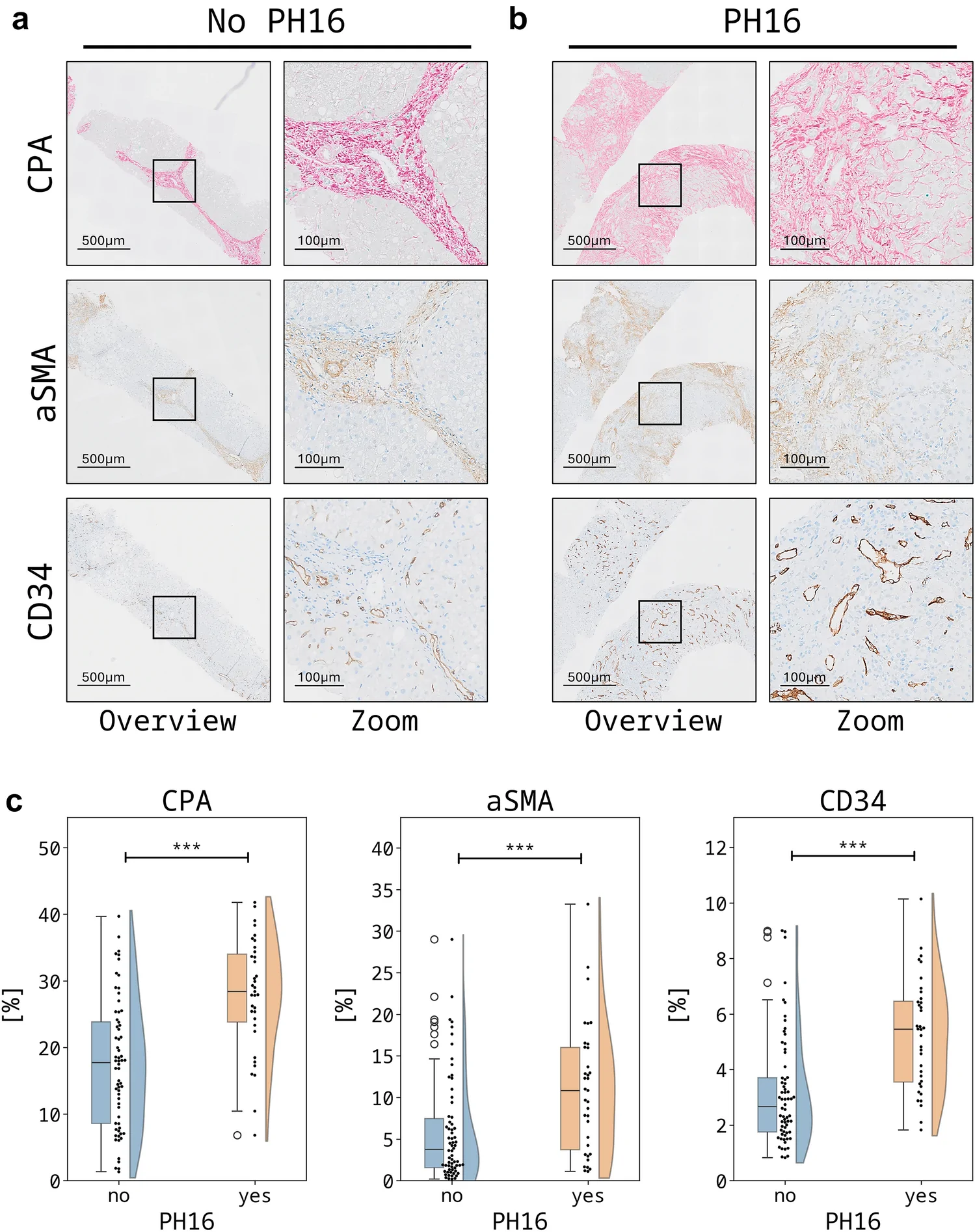
Comparison of histological biomarkers by PH severity. Representative images of picrosirius-red (top row), alpha-smooth-muscle-actin (middle row), and CD34 (bottom row) stains in (**a**) a patient without PH16 and (**b**) with PH16. Sharpness was enhanced for visualization purposes. **c** All histological biomarkers displayed a significant increase in positive area in patients with PH16. The p-values were calculated with Mann–Whitney-U test. *αSMA* alpha-smooth-muscle-actin, *CPA* collagen proportionate area, *PH16* HVPG ≥ 16 mmHg

We assessed the expression of all three histological biomarkers based on PH severity, namely CPA as a marker of the static component of PH, as well as αSMA and CD34 as dynamic markers of PH. Comparisons were made in patients without PH16 (Fig. 1a) and with PH16 (Fig. 1b). We detected a significantly higher CPA in the PH16 group (PH16: 28.39 [23.82–34.00] %, vs. no PH16: 17.72, [8.58–23.87] %; p < 0.001, Fig. 1c). Similarly, αSMA, a marker of myofibroblast activation, was significantly elevated in the PH16 group (PH16: 10.84, [3.71–16.00] %, vs. no PH16: 3.75, [1.56–7.47] %; p < 0.001). CD34, a marker of sinusoidal capillarization, also showed a robust increase in PH16 (PH16: 5.45, [3.55–6.46] % vs. no PH16: 2.67, [1.75–3.71] %; p < 0.001). We next tested whether similar effects could be observed when differentiating by fibrosis stage (Supplementary Fig. 1A). All three biomarkers were significantly enriched in F4 fibrosis compared to ≤ F3, with strong effects observed for all biomarkers (CPA: 25.36 [17.96–31.14] % vs. 11.66 [6.88–18.07] %, p < 0.001; αSMA: 7.41 [2.34–14.47] % vs. 2.54 [1.13–4.91] %, p < 0.001; CD34: 3.76 [2.80–6.27] % vs. 2.14 [1.53–3.28] %, p < 0.001). Stratified by compensation status (Supplementary Fig. 1B), we detected a significant difference in the expression of CD34 (decompensated: 4.59 [2.98–5.88] % vs. compensated: 2.47 [1.71–3.66] %; p < 0.001), and to a lesser extent CPA (decompensated: 25.36 [17.48–31.14] %, vs. compensated: 17.40 [8.80–25.35] %; p = 0.003).

### Histological biomarkers correlate with surrogates of portal hypertension severity

**Fig. 2:**
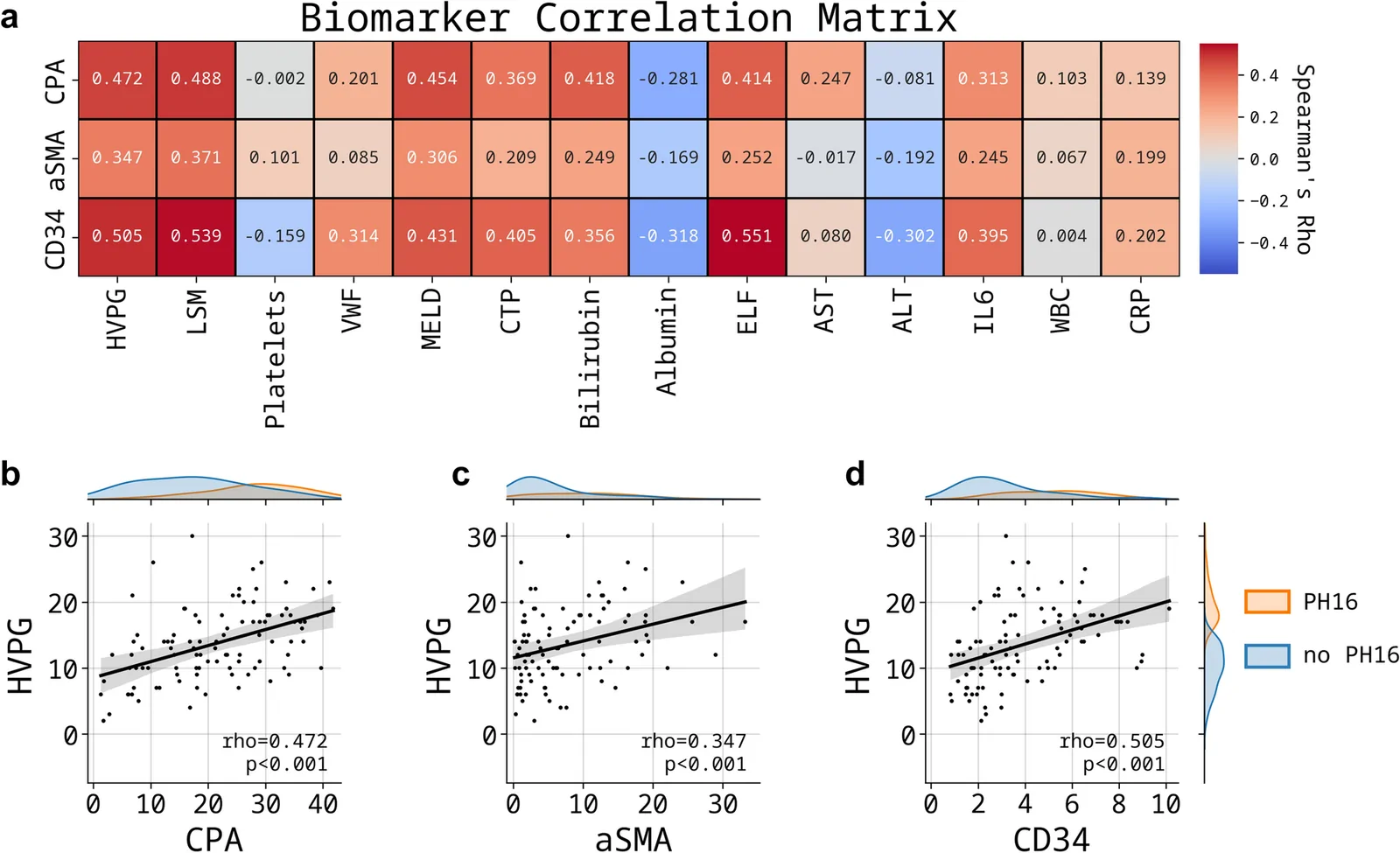
Correlation of histological biomarkers with PH-related parameters. **a** Correlation matrix of all three histological biomarkers (CPA, αSMA, CD34) with diverse clinical parameters of portal hypertension. For all comparisons, Spearman’s rho coefficients were calculated. **b**–**d** Scatterplots of **b** CPA **c** αSMA, and **d** CD34 vs. HVPG. Shaded areas indicate the 99% CI. *ALT* alanine aminotransferase, *AST* aspartate aminotransferase, *CI* confidence interval, *CPA* collagen proportionate area, *CRP* C-reactive protein, *CTP* Child-Turcotte-Pugh Stage, *ELF* enhanced liver fibrosis score, *HVPG* hepatic venous pressure gradient, *IL6* interleukin 6, *LSM* liver stiffness measurement, *MELD* model for end-stage liver disease, *PH16* HVPG ≥ 16 mmHg, *VWF* von Willebrand factor, *WBC* white blood cells, *αSMA* lpha-smooth-muscle-actin

We next assessed how histological biomarkers correlate with clinical parameters related to PH (HVPG, LSM, platelets, VWF), liver function (model for end-stage liver disease [MELD] score, Child-Turcotte-Pugh [CTP] score, bilirubin, albumin), and liver injury and inflammation (ELF, aspartate aminotransferase, alanine aminotransferase, IL6 and CRP; Fig. 2a). Overall, CD34 showed the highest correlation with these parameters. CD34 showed a moderate correlation with LSM (rho = 0.539, p < 0.001), MELD score (rho = 0.431, p < 0.001) and ELF (rho = 0.551, p < 0.001). Notably, CD34 also showed a positive correlation with VWF (rho = 0.314, p = 0.002), which was not observed for CPA and αSMA. In contrast, when compared to αSMA and CD34, CPA correlated better with bilirubin (rho = 0.418, p < 0.001) and had a similar correlation with MELD score (rho = 0.454, p < 0.001) compared to CD34. CPA further also showed a relevant correlation with LSM (rho = 0.488, p < 0.001). While the overall correlation of αSMA was lower, αSMA showed its highest associations with LSM (rho = 0.371, p < 0.001), MELD-score (rho = 0.306, p = 0.002), ELF (rho = 0.252, p = 0.015) and a weak association with two markers of inflammation (αSMA vs. IL6: rho = 0.245, p = 0.017; vs. CRP: rho = 0.199, p = 0.047). However, the αSMA correlations remained weaker than the ones observed for CPA and CD34.

The overall trend was confirmed when comparing the histological biomarkers to HVPG values (Fig. 2b–d). CD34 showed the highest correlation (rho = 0.505, p < 0.001; Fig. 2d), followed by CPA (rho = 0.472, p < 0.001; Fig. 2b) and αSMA (rho = 0.347, p < 0.001; Fig. 2c). To assess whether etiology impacts the correlation of these biomarkers with HVPG, we additionally performed a subgroup analysis (Supplementary Table 1). CD34 achieved a very high correlation with HVPG in metabolic dysfunction-associated steatotic liver disease patients (rho = 0.611, p < 0.001) but was comparatively weaker in other etiologies. CPA achieved numerically higher correlations than CD34 and αSMA in viral hepatitis (rho = 0.416, p = 0.204) and cholestatic liver disease (rho = 0.407, p = 0.167), although these results were not significant. In the alcohol-related liver disease subgroup, correlations were weaker and comparable among histological biomarkers. However, these results should be interpreted with caution due to overall low subgroup sample sizes.

We additionally assessed whether the histological biomarkers show relevant dynamics with each other (Supplementary Fig. 2A–C). Indeed, CPA correlated strongly with both αSMA (rho = 0.609, p < 0.001) and CD34 (rho = 0.718, p < 0.001), while the correlation between αSMA and CD34 was weaker (rho = 0.579, p < 0.001). This suggests that all three biomarkers share a common histological effect axis but may still carry different information content.

### Analysis of dynamic components of portal hypertension orthogonal to fibrosis

**Fig. 3:**
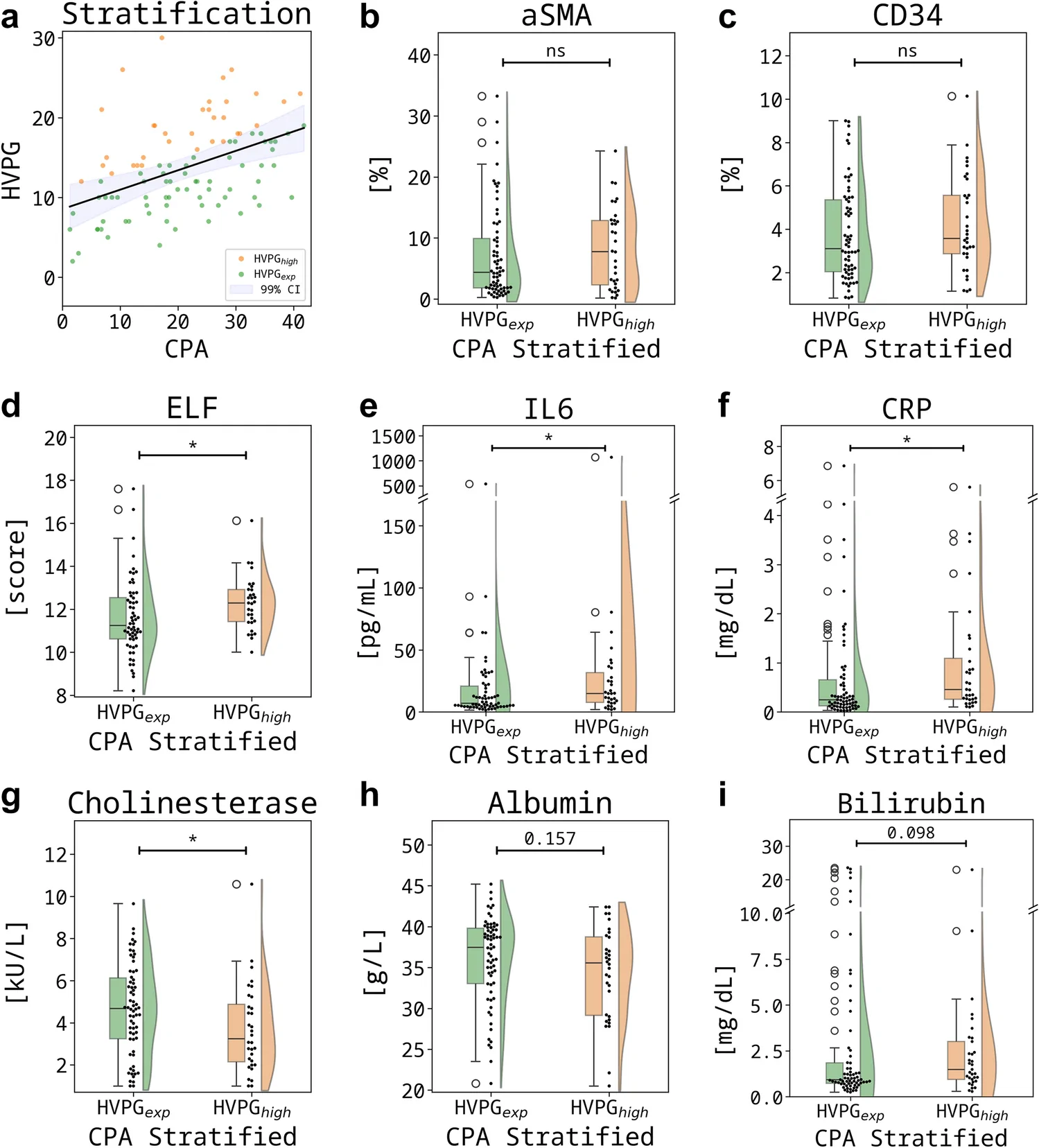
Biomarker surrogates of portal hypertension orthogonal to the static component. **a** Stratification into patients with expected (HVPG_exp_, n = 66) and higher than expected (HVPG_high_, n = 33) HVPG based on the linear regression of HVPG on CPA. Strata were defined based on the 99% CI. **b** αSMA and **c** CD34 were unchanged in HVPG_high_ patients. **d** ELF score showed an increase from HVPG_exp_ to HVPG_high_. **e** IL6 also increased from HVPG_exp_ to HVPG_high_. **f** CRP showed an increase in HVPG_high_. **g** Cholinesterase was significantly decreased in the HVPG_high_ stratum, whereas (**h**) albumin showed a trend for lower values. **i** Bilirubin remained unchanged. *CPA* collagen proportionate area, *CRP* C-reactive protein, *ELF* enhanced liver fibrosis score, *HVPG* hepatic venous pressure gradient, *IL6* interleukin 6, *αSMA* alpha-smooth-muscle-actin

The static component of PH, as reflected by histological CPA and non-invasive LSM assessment, is a major driver of PH. Regardless, many patients may display a higher-than-expected HVPG based on the static component quantification. To assess which clinical parameters might be associated with these deviations, we stratified patients into two groups (Fig. 3a): expected HVPG (HVPG_exp_, n = 66) and higher-than-expected HVPG (HVPG_high_, n = 33). Patients in the HVPG_high_ group did not significantly differ from patients in the HVPG_exp_ group in terms of histological biomarker expression (Fig. 3b, c), although both αSMA and CD34 had discreetly elevated median values in the HVPG_high_ group. From HVPG_exp_ to HVPG_high_, we detected increases in three clinical biomarkers (Fig. 3d–f). The ELF score was significantly higher in HVPG_high_ patients compared to HVPG_exp_ (HVPG_high_: 12.29 [11.42–12.91] vs. HVPG_exp_: 11.25 [10.61–12.55], p = 0.020, Fig. 3d). IL6, a marker of inflammation, showed an increase in HVPG_high_ (HVPG_high_: 14.90 [7.64–31.64] pg/mL vs. HVPG_exp_: 6.91 [4.42–20.90] pg/mL, p = 0.048, Fig. 3e). CRP, another marker of inflammation, also showed an increase in HVPG_high_ patients (HVPG_high_: 0.46 [0.26–1.09] mg/dL, vs. HVPG_exp_: 0.25 [0.12–0.66] mg/dL, p = 0.014; Fig. 3f). Lastly, cholinesterase (HVPG_high_: 3.24 [2.14–4.87] kU/L vs. HVPG_exp_: 4.68 [3.24–6.13] kU/L, p = 0.035), a clinical biomarker of liver function, was significantly decreased in the HVPG_high_ stratum (Fig. 3g), whereas albumin showed only a trend for decreased values (p = 0.157; Fig. 3h) and bilirubin remained unchanged (Fig. 3i).

To assess potential dynamic effects of αSMA and CD34 while partially controlling for fibrosis severity, we additionally stratified patients based on CPA tertiles (n = 33 each, Supplementary Fig. 3A): CPA values from 1.32–16.55% (tertile 1, T1), CPA values from 16.55 to 26.48% (tertile 2, T2) and CPA values from 26.48 to 41.78% (tertile 3, T3). Within each tertile, patients were further sub-stratified based on the within-tertile median αSMA or CD34 value into a below and above-median group. While not statistically significant, we observed that patients with higher αSMA in the T1 CPA stratum tended to have higher HVPG values (αSMA-high: 12 mmHg vs. αSMA-low: 8 mmHg; p = 0.129; Supplementary Fig. 3B). For CD34, in addition to a numerically higher HVPG in the T1 CPA stratum (p = 0.129), we also detected a nominally higher HVPG in the T2 CPA stratum (CD34-high: 15.5 mmHg vs. CD34-low: 12 mmHg; p = 0.035; Supplementary Fig. 3. In T3, i.e. the most severe stratum, neither αSMA nor CD34 separated patients by HVPG value.

**Fig. 4:**
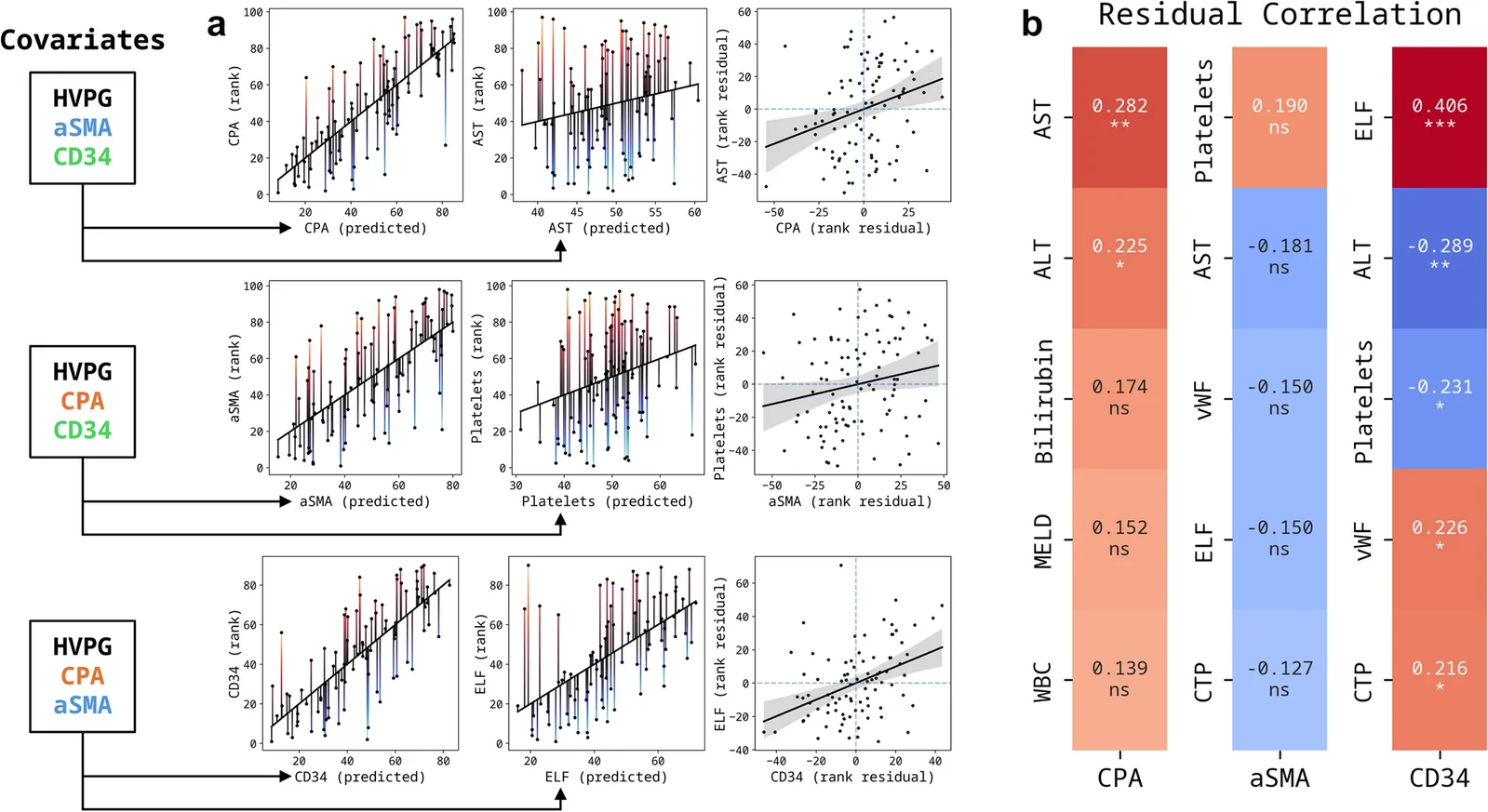
Partial correlation analysis of histological biomarkers. **a** Multivariate Spearman-based partial correlation analysis was performed to assess the independent correlation of histological biomarkers to clinical parameters. Each histological biomarker and clinical parameter was rank-transformed and residualized against the same covariate set, consisting of HVPG and the remaining two histological biomarkers. The rank residuals between true value and predicted value were taken as the portion of the variable unexplained by covariates. **b** Residual to residual Spearman correlation of histological biomarkers with clinical parameters. αSMA did not show significant correlations with any of the measured clinical parameters. CD34, instead, correlated independently with ELF, linking it more closely to sinusoidal remodeling and perisinusoidal fibrogenesis. Other notable correlations were between CD34 and platelets, as well as VWF. CPA independently correlated with liver injury markers AST and ALT. *ALT* alanine aminotransferase, *AST* aspartate aminotransferase, *CPA* collagen proportionate area, *CRP* C-reactive protein, *ELF* enhanced liver fibrosis score, *HVPG* hepatic venous pressure gradient, *VWF* von Willebrand factor, *WBC* white blood cell, *αSMA* alpha-smooth-muscle-actin

We further assessed the independent correlation of each histological biomarker to clinical parameters using multivariate partial correlation (Fig. 4a). Each respective histological biomarker was rank-transformed and fitted as a dependent variable using multivariate linear regression, with HVPG and the remaining two histological biomarkers serving as independent variables. The same was performed on the clinical parameters of interest and the remaining rank residuals between predicted and true value were correlated against each other (Fig. 4b). CPA residuals correlated significantly with residuals of liver injury markers asparate aminotransferase (rho = 0.282, p = 0.006) and alanine aminotransferase (rho = 0.225, p = 0.029). αSMA residuals did not have a significant independent correlation with any of the measured biomarkers but showed a weak correlation with platelet residuals (rho = 0.190, p = 0.065). Lastly, CD34 residuals had a significant correlation with ELF (rho = 0.406, p < 0.001), in addition to inverse correlations with alanine aminotransferase (rho = − 0.289, p = 0.005) and platelet count (rho = − 0.231, p = 0.024). We also observed a significant independent correlation with VWF (rho = 0.226, p = 0.030). Due to the significant independent association between CD34 and ELF, we additionally performed a correlation analysis between all three histological biomarkers and the three ELF components (tissue inhibitor of metalloproteinases 1, TIMP1; amino-terminal propeptide of type III collagen, P3NP; hyaluronic acid, HA; Supplementary Table 2). CD34 showed the highest correlation with HA (rho = 0.508, p < 0.001), followed by P3NP (rho = 0.467, p < 0.001) and TIMP1 (rho = 0.404, p < 0.001), whereas CPA and αSMA showed the numerically highest correlations with TIMP1 (CPA: rho = 0.372, p < 0.001; αSMA: rho = 0.322, p < 0.001).

### Benchmarking Histological Biomarkers for Diagnosis of Portal Hypertension

**Fig. 5:**
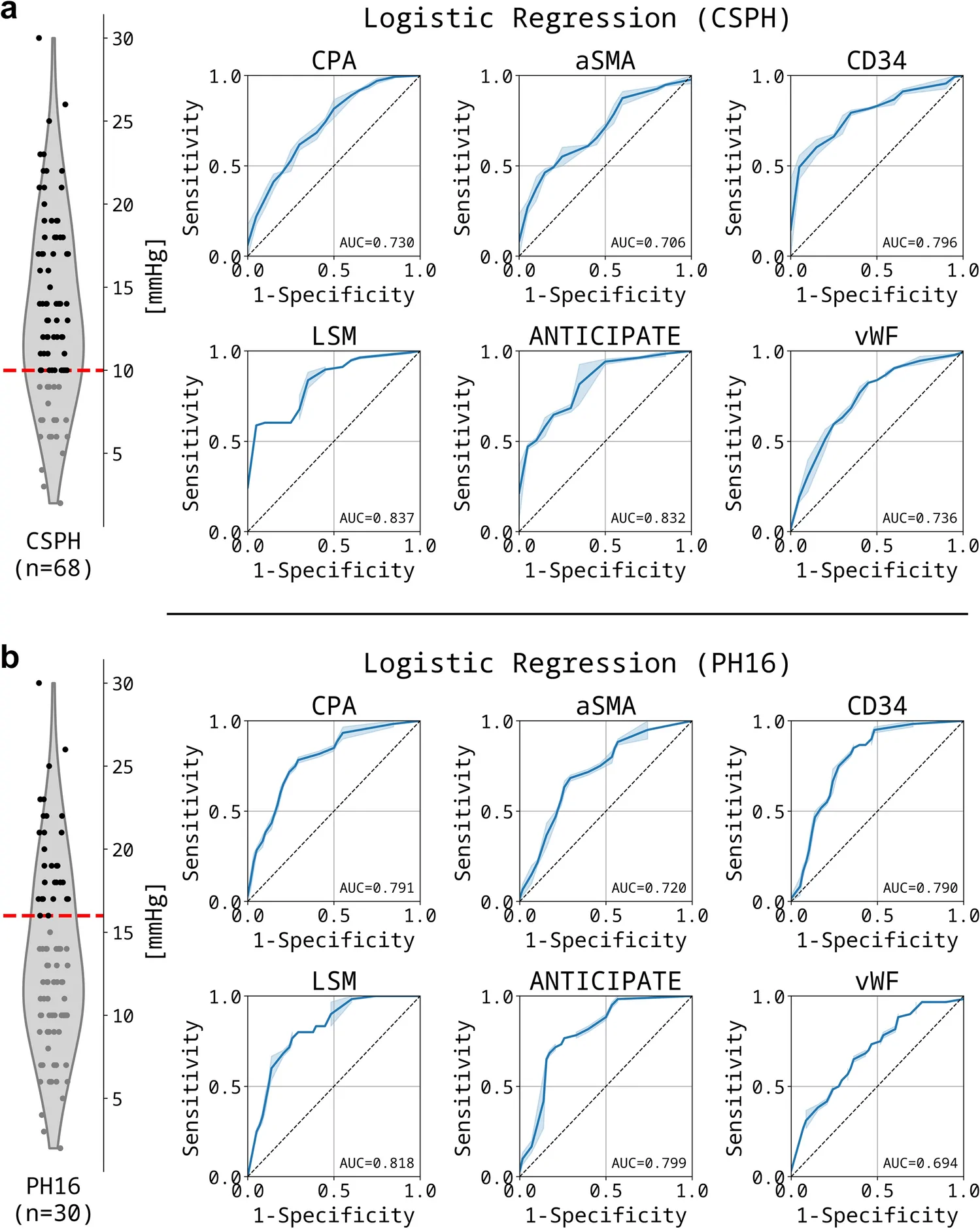
Comparison of predictive performance for CSPH and PH16 between histological biomarkers and non invasive testss. **a** Across all three histological biomarkers, CD34 showed the best performance for predicting CSPH with an AUROC of 0.796. However, CD34 only outperformed VWF for prediction of CSPH, with the best performance observed for LSM with an AUROC of 0.837. **b** CPA had higher performance for PH16 prediction compared to CSPH but remained comparable with CD34. However, they still did not fully outperform non invasive tests. *CPA* collagen proportionate area, *CSPH* clinically significant portal hypertension, *HVPG* hepatic venous pressure gradient, *LSM* liver stiffness measurement, *PH16* HVPG ≥ 16 mmHg, *VWF* von Willebrand Factor, *αSMA* alpha smooth-muscle-actin

We explored whether the three histological biomarkers contain discriminative potential for common clinical PH severity strata and additionally compared them to common non-invasive tests in a complete-case subset of the cohort (n = 88). We performed univariate logistic regression to predict CSPH (Fig. 5a) and PH16 (Fig. 5b). CD34 outperformed both CPA and αSMA for the prediction of CSPH with an AUROC of 0.796. Using Youden’s index, we determined the optimal cut-off for CSPH prediction as 3.58%, which yielded a sensitivity of 0.559 and specificity of 0.950. In contrast, the AUROC of αSMA was 0.706, with a sensitivity of 0.603 and specificity of 0.750 at a cut-off of 5.06%. Lastly, CPA had a higher sensitivity (0.868) and low specificity (0.500) at a cut-off of 12.31% (AUROC = 0.730). However, none of the histological biomarkers outperformed LSM (AUROC = 0.837) and ANTICIPATE (AUROC = 0.832), with only VWF (AUROC = 0.736) having a lower predictive performance than CD34.

For prediction of PH16, CD34 (AUROC = 0.790, Sensitivity = 0.867, Specificity = 0.638, cut-off = 3.12%) and CPA (AUROC = 0.791, Sensitivity = 0.800, Specificity = 0.707, cut-off = 22.41%) had comparable performance, followed by αSMA (AUROC = 0.720, Sensitivity = 0.667, Specificity = 0.741, cut-off = 7.79%). However, LSM (AUROC = 0.818) and ANTICIPATE (AUROC = 0.799) still had better predictive performance.

## Discussion

In this cross-sectional cohort of CLD patients of different liver disease etiologies well-characterized for PH severity, we used digitized histomorphometry to systematically evaluate the importance of static (CPA) and dynamic (αSMA, CD34) histological biomarkers to PH pathomechanisms. All three biomarkers showed a strong increase in patients suffering from severe portal hypertension. While CPA was reaffirmed as an established surrogate of the static component of PH, CD34, a marker of sinusoidal capillarization, emerged as a robust surrogate of overall PH severity. Importantly, its independent association with ELF and VWF after adjustment for HVPG, CPA and αSMA underline the potential value of CD34, ELF and VWF as sinusoidal capillarization biomarkers. In contrast, despite αSMA being markedly elevated in PH16 patients, it correlated weakly with the time-matched HVPG, suggesting it may capture a separate facet of the portal hypertensive syndrome.

Surrogates of the static and dynamic components of PH are valuable as they inform our understanding of PH pathophysiology [9, 25]. CPA was previously applied for histologic estimation of PH severity via the static component [19, 20], but the methodology is limited to quantification of already deposited collagenous fibers in the tissue. CPA provides valuable insight into fibrosis severity, but the inherent gradual and long-term course of fibrosis development may limit the potential of CPA to reflect dynamic fibrosis trends [3]. Moreover, a previous study has shown that in more severe PH, the link between HVPG and the static component, measured via LSM, weakens [26]. We therefore hypothesized that CD34 and αSMA might act as more responsive dynamic markers, since both reflect cellular processes which shift more rapidly than the evaluation of deposited collagen.

αSMA marks the activation of quiescent HSCs into contractile, fibrosis producing myofibroblasts [12, 27]. This state can both intensify and resolve over the disease course [3]. Because myofibroblast activation is reversible, αSMA could potentially track the current fibrogenic trend rather than cumulative fibrosis itself, including the well-established phenomenon of liver fibrosis regression [3]. This is also supported by functional studies in pre-clinical liver fibrosis models which show decline in hepatic αSMA area or αSMA expression when diseased rodents are treated with anti-fibrotic agents [28]. Sinusoidal capillarization, as reflected by CD34 [5], represents another important functional aspect of fibrogenesis and PH [29]. An increase in CD34-positive area has been documented in diseased pre-clinical models of liver fibrosis [30], as well as in patients with hepatocellular carcinoma, where intratumoral capillarization is a recognized phenomenon [31]. A recent study suggested clinical relevance of capillarization in patients with metabolic dysfunction-associated steatotic liver disease, since the density of CD34-positive vessels gradually increases from liver fibrosis stages 0–3 [30]. The same study also concluded that patients treated with lanifibranor, a pan-PPAR agonist, showed significant improvements in the periportal CD34-positive vessel density [30].

In our study, we found that both αSMA and CD34 were significantly higher expressed in established F4 cirrhosis compared to ≤ F3 fibrosis stages. Importantly, we expanded this finding by investigating the pathomechanistic link of αSMA and CD34 to PH severity – as measured by HVPG. We established that patients with PH16 show even higher histological expression of all three biomarkers than patients without pronounced PH (i.e., HVPG < 16 mmHg). We further assessed the overall correlation of all histological biomarkers with a diverse set of clinical parameters related to PH. CD34 had a higher correlation with HVPG (rho = 0.505) compared to CPA (rho = 0.472) and αSMA (rho = 0.347). The comparatively lower correlation of αSMA indicates that αSMA is more decoupled from the time-matched HVPG values, which is in-line with a role as a dynamic fibrogenesis indicator, rather than a surrogate of PH severity. This was also observed on a tissue level, as αSMA correlated less tightly with both CPA and CD34 than those two biomarkers did with each other. While our cross-sectional, HVPG-centered cohort cannot confirm αSMA as an “on/off” compass of disease trajectory, future studies should focus on exploring the potential of αSMA for the prediction of short-term disease outcomes rather than PH severity.

The higher association of CD34 with HVPG was further underlined by its higher diagnostic value for prediction of CSPH (AUROC = 0.796) and comparable AUROCs for prediction of PH16 (AUROC = 0.790) compared to CPA. While this serves as valuable confirmatory evidence, the effects are insufficient to justify clinical utility, and none of the invasive histological biomarkers outperformed widely available non invasive tests like LSM and ANTICIPATE score. Importantly, αSMA and CD34 should be positioned as valuable mechanistic readouts, rather than diagnostic tools, and could provide additive information on pathomechanistic drivers within the portal hypertensive syndrome.

We also identified a significant correlation between CD34 and ELF, a marker of extracellular matrix remodeling. This result remained significant on analysis adjusted for HVPG, αSMA and CPA, and points towards a pathomechanistic cellular cross-talk between LSECs and HSCs, where capillarization of LSECs represents an early event in the disease course of CLD which is connected to loss of the quiescent HSC phenotype [32]. However, this result may suggest that ELF also reflects endothelial dysfunction, as two of three ELF score components – namely P3NP and HA – are cleared by LSECs [33–36]. Dysfunctional LSECs have a reduced clearance capacity [37], thereby potentially contributing to increased systemic levels of those two ELF components next to increased matrix turnover. Indeed, CD34 showed a higher correlation with ELF components HA and P3NP, whereas CPA and αSMA both had numerically higher correlations with the ELF component TIMP1. We also identified an independent correlation between CD34 and VWF, an established biomarker of endothelial cell dysfunction [38] and a versatile surrogate for PH severity and prognosis [39–42]. This supports the evidence that CD34 positive capillarized LSECs may contribute to systemic VWF levels.

To identify clinical factors associated with portal pressure beyond what is explained by the static component, we stratified patients into those with expected (HVPG_exp_) and higher-than-expected (HVPG_high_) HVPG relative to their CPA. While the histological biomarkers showed numerically higher HVPG values in the HVPG_high_ stratum, this result was not statistically significant, which could reflect their shared histological axis with CPA. In line with previous studies [13], we found that circulating biomarkers, such as ELF (reflecting extracellular matrix remodeling and potentially endothelial dysfunction), CRP and IL6 (reflecting inflammation) were significantly increased in HVPG_high_ patients, while cholinesterase (reflecting hepatic function) was decreased. To assess whether αSMA and CD34 nonetheless carry dynamic information once fibrosis severity is partially controlled for, we sub-stratified patients within CPA tertiles by median biomarker expression. A signal for both histological biomarkers was observed in the lower fibrosis strata: higher αSMA and CD34 each marked higher HVPG values in the lower fibrosis tertile, in addition to CD34 significantly marking higher HVPG in the middle tertile (p = 0.035). However, in the higher fibrosis tertile, neither separated patients by HVPG. Importantly, these analyses are exploratory and likely underpowered to establish dynamic effects, but they support a role of both biomarkers as disease indicators and should motivate more granular study designs to confirm these findings.

Our study also has limitations: First, our cross-sectional cohort of 102 CLD patients is moderately sized, and a larger cohort could increase statistical power and strengthen the findings. A longitudinal study design would allow a more granular characterization of the dynamic PH component. However, the cohort was thoroughly characterized for PH severity by HVPG and a wide set of pathophysiologic biomarkers and was sufficiently sized to obtain statistically significant and clinically relevant findings. Further, we cannot exclude operator and sampling bias, which are inherent to liver biopsy acquisition and analysis. We controlled for both factors through an objective annotation strategy, which aimed at removing larger areas of parenchyma-free connective tissue and mechanically damaged areas. Our approach in CD34 quantification differed from previous studies [30]. While other studies focused on quantifying CD34 positive vessel densities in the midlobular zone, we quantified CD34 as the total positive area of the whole tissue. This approach was chosen under the assumption that total area quantification is sufficient to capture pathological CD34 changes, like expression within fibrosis and the midlobular parenchymal area, compared to healthy baselines. Lastly, stains were performed in different batches, and batch effects cannot be excluded.

In conclusion, CD34 and αSMA may represent valuable histologic surrogates of sinusoidal capillarization and myofibroblast activation in CLD patients, capturing distinct dimensions of PH severity. Importantly, the sinusoidal capillarization marker CD34 was closely linked to PH severity, and specifically also to ELF and VWF as more readily available circulating biomarkers of sinusoidal remodeling, perisinusoidal fibrogenesis and LSEC activation. αSMA was more decoupled from HVPG severity, consistent with a role in tracking active fibrogenesis rather than accumulated fibrosis. Future studies should investigate the prognostic value and longitudinal association with the dynamic PH component of histologic αSMA, CD34 and blood-based circulating VWF for the prediction of further clinical course of patients with CLD.

## Supplementary Information

Below is the link to the electronic supplementary material.

Supplementary file1 (DOCX 1110 KB)

## Data Availability

Data can be provided upon reasonable request to the corresponding author.

## Abbreviations

CI: Confidence interval
CLD: Chronic liver disease
CPA: Collagen-proportionate area
CRP: C-reactive protein
CSPH: Clinically significant portal hypertension
CTP: Child-Turcotte-Pugh
ELF: Enhanced liver fibrosis score
HA: Hyaluronic acid
HSC: Hepatic stellate cell
HVPG: Hepatic venous pressure gradient
HVPG_exp_: Expected hepatic venous pressure gradient
HVPG_high_: Higher-than-expected hepatic venous pressure gradient
IL6: Interleukin 6
LSEC: Liver sinusoidal endothelial cell
LSM: Liver stiffness measurement
MELD: Model for end-stage liver disease
PH: Portal hypertension
PH16: Hepatic venous pressure gradient ≥ 16 mmHg
P3NP: Amino-terminal propeptide of type III collagen
T: Tertile
TIMP1: Tissue inhibitor of metalloproteinases 1
VWF: Von Willebrand factor
WBC: White blood cells
αSMA: Alpha-smooth-muscle-actin
