# Supplementary material for: Histomorphometry indicates distinct contributions of myofibroblast activation and sinusoidal capillarization to portal hypertension
Source: Hepatol Int. 2026 Jul 27;20(5):1432–45. doi: 10.1007/s12072-026-11133-0 (PMC13676638; doi:10.1007/s12072-026-11133-0)
Supplement: Supplementary file 1 [file 12072_2026_11133_MOESM1_ESM.docx]

**Supplementary Materials**

**Histomorphometry indicates distinct contributions of myofibroblast activation and sinusoidal capillarization to portal hypertension**

Thomas SORZ-NECHAY, MD^1,2,3,4^

Benedikt S. HOFER, MD^1,2,3,4^

Philipp KÖNIGSHOFER, DVM^1,2,3^

Alexis TREITLER^1,2^

Georg SEMMLER, MD PhD^1,4^

Benedikt SIMBRUNNER, MD, PhD^1,2,4^

Vlad TARU, MD^1,2^

Katharina BONITZ, MSc^1,2,3^

Henriette HORSTMEIER, MD^1^

Georg KRAMER, MD^1,2,3,4^

Kerstin ZINOBER^1,2,4^

Katharina REGNAT^1,2,4^

Mattias MANDORFER, MD PhD^1,4^

Barbara NEUDERT^5^

Christopher C. KALTENECKER^5^

Behrang MOZAYANI, MD^5^

Renate KAIN, MD^5^

Michael TRAUNER, MD^1,4^

Philipp SCHWABL, MD PhD^1,2,3,4^

Thomas REIBERGER, MD^1,2,3,4*^

^1^ Division of Gastroenterology and Hepatology, Department of Medicine III, Medical University of Vienna, Vienna, Austria.

^2^ Christian Doppler Laboratory for Portal Hypertension and Liver Fibrosis, Medical University of Vienna, Vienna, Austria.

^3^ CeMM Research Center for Molecular Medicine of the Austrian Academy of Sciences, Vienna, Austria.

^4^ Clinical Research Group MOTION, Medical University of Vienna, Vienna, Austria

^5^ Department of Pathology, Medical University of Vienna, Vienna, Austria

***Corresponding author**: Thomas REIBERGER, MD

Division of Gastroenterology and Hepatology

Department of Medicine III

Währinger Guertel 18-20

A-1090 Vienna, Austria

P: +4314040065890

F: +4314040047350

M: [thomas.reiberger@meduniwien.ac.at](mailto:thomas.reiberger@meduniwien.ac.at)

**Supplementary Methods**

**Histological Staining**

All liver biopsy specimens were formalin fixed (7.5% neutral buffered formaldehyde), paraffin-embedded and cut at 2μm thickness.

For picrosirius-red/fast-green staining, unstained tissue sections were deparaffinized in xylene and rehydrated in descending concentrations of ethanol (99%, 80%, 70%, 60%) for 5 minutes each. Following staining with “Weigerts Eisenhämatoxylin” (#HT1079, Sigma-Aldrich, Missouri, United States) according to the manufacturer’s instructions, the tissue was stained in 0.1% Fast Green (#16596, Morphisto, Offenbach am Main, Germany) for 4 minutes, briefly rinsed in acetic acid and deionized water and immersed in 0.1% picrosirius-red solution (#13425, Morphisto, Offenbach am Main, Germany) for 1 hour. Lastly, the tissue was dehydrated in ascending concentrations of ethanol, followed by immersion in xylene and covered in hydrophobic mounting medium.

Binding of antibodies to alpha alpha-smooth-muscle-actin (αSMA: mouse monoclonal antibody to Actin, Smooth Muscle [1A4], #760-2833 IVD, ready-to-use, CellMarque, California, USA; CD34: mouse monoclonal antibody to CD34 [QBEnd/10], #NCL-L-END, dilution 1:50, Leica Biosystems, Nussloch, Germany) after enzymatic antigen retrieval were visualized by automated immunohistochemistry using a Novolink^TM^ Polymer Detection system on an Immunostainer Benchmark Ultra (Ventana/Roche). Following dehydration in ascending concentrations of ethanol and immersion in xylene, samples were covered in hydrophobic mounting medium.

**Histomorphometry Pipeline**

All slides were digitized in high-resolution (40x objective, 0.137μm/pixel) using the Olympus SLIDEVIEW VS200 research slide-scanner (EVIDENT Europe GmbH, Hamburg, Germany). Digital histomorphometry was performed using customized computational pipelines. First, all images were converted to .tiff format at a downscaled resolution (20x, 0.274μm/pixel) using bfconvert (version 5.6.0). If multiple layers of the same section were available on a slide, the highest quality section was chosen and cropped from the image. Damaged tissue regions, artifacts and large areas of parenchyma-free connective tissue were marked using QuPath (version 0.5.1), exported as masks in portable network graphics (.png) format, and masks were used in Python for exclusion.

The pre-processed images were then analyzed with a customized pipeline. Briefly, images were converted to Hue-Saturation-Lightness (HSL) color space, denoised using Gaussian-Blur with a kernel-size of 75x75 pixels and tissue was detected via thresholding (Lightness-Channel pixel-value < 235). In samples with faint staining or strong background intensity, the lightness threshold was adjusted on a per-case basis. If no suitable lightness threshold could be found, the tissue was segmented manually in QuPath. The resulting tissue regions were filtered using area-based exclusion (<0.1mm^2^) to remove artifacts.

Following tissue segmentation, quantification of the histological biomarker was performed by converting the image to Hue-Saturation-Value color space, applying Gaussian-Blur with a kernel-size of 5x5 pixels and performing color segmentation based on empirically determined thresholds. For immunohistochemical stains, diaminobenzidine area was quantified using the following thresholds: 80-140 (Hue), 25-250 (Saturation), 5-200 (Value). For picrosirius-red stains, collagen proportionate area (CPA) was quantified using the following thresholds: 110-160 (Hue), 30-210 (S), 190-255 (V). The biomarker area was calculated as the number of positive pixels (color mask) divided by the total tissue pixels (tissue mask), expressed as percentage.

**Supplementary Figures**

**Supplementary Figure 1: Histological Biomarkers by Fibrosis Stage and Decompensation Status**. Abbreviations: CPA - collagen proportionate area, αSMA - alpha smooth-muscle-actin.

**Supplementary Figure 2: Shared effects of Histological Biomarkers.** Abbreviations: CPA - collagen proportionate area, αSMA - alpha smooth-muscle-actin.

**Supplementary Figure 3: Dynamic effects of αSMA and CD34 on HVPG after controlling for fibrosis severity.** (A) Representative violinplot of CPA severity strata. The x-axis has been warped for visualization purposes. (B) HVPG values after sub-stratification based on within-tertile median αSMA. Patients in the T1 CPA tertile showed a trend for higher HVPG values if αSMA was higher. (C) HVPG values after sub-stratification based on within-tertile median CD34. Patients in the T1 CPA tertile showed a trend for higher HVPG values if CD34 was higher, in addition to a significant difference in the T2 CPA tertile. Abbreviations: CPA - collagen proportionate area, HVPG – hepatic venous pressure gradient, T – tertile, αSMA - alpha smooth-muscle-actin.

**Supplementary Tables**

| Etiology | n | Histological Biomarker | Spearman's rho | p-value |
| --- | --- | --- | --- | --- |
| ALD | 32 | CPA | 0.224 | 0.217 |
|  |  | CD34 | 0.222 | 0.222 |
|  |  | αSMA | 0.165 | 0.368 |
| Cholestatic | 14 | CPA | 0.407 | 0.167 |
|  |  | CD34 | 0.248 | 0.393 |
|  |  | αSMA | 0.027 | 0.928 |
| MASLD | 28 | CPA | 0.423 | 0.025 |
|  |  | CD34 | 0.611 | 0.001 |
|  |  | αSMA | 0.465 | 0.014 |
| Viral | 11 | CPA | 0.416 | 0.204 |
|  |  | CD34 | 0.201 | 0.554 |
|  |  | αSMA | 0.105 | 0.759 |

**Supplementary Table 1: Subgroup analysis of HVPG correlation to histological biomarkers by etiology**. MetALD (n=7) patients were merged with MASLD. Abbreviations: ALD – alcohol-related liver disease, CPA – collagen proportionate area, MASLD – metabolic dysfunction-associated steatotic liver disease, αSMA - alpha smooth-muscle-actin.

| Independent Variable | Dependent Variable | Spearman's rho | p-value |
| --- | --- | --- | --- |
| CPA | TIMP1 | 0.372 | p<0.001 |
|  | P3NP | 0.299 | p=0.003 |
|  | HA | 0.341 | p<0.001 |
| αSMA | TIMP1 | 0.322 | p=0.001 |
|  | P3NP | 0.201 | p=0.054 |
|  | HA | 0.202 | p=0.052 |
| CD34 | TIMP1 | 0.404 | p<0.001 |
|  | P3NP | 0.467 | p<0.001 |
|  | HA | 0.508 | p<0.001 |

**Supplementary Table 2: Correlation of histological biomarkers to ELF score components**. Abbreviations: HA – hyaluronic acid, P3NP – aminoterminal peptide of type III collagen, TIMP1 – tissue inhibitor of metalloproteinase 1.
